# Kitaoka’s Tomato: Two Simple Explanations Based on Information in the Stimulus

**DOI:** 10.1177/2041669517749601

**Published:** 2018-01-08

**Authors:** Arthur Shapiro, Laysa Hedjar, Erica Dixon, Akiyoshi Kitaoka

**Affiliations:** Department of Psychology, American University, Washington, DC, USA; Department of Computer Science, American University, Washington, DC, USA; Program in Behavior, Cognition, and Neuroscience, American University, Washington, DC, USA; Program in Behavior, Cognition, and Neuroscience, American University, Washington, DC, USA; Department of Psychology, Ritsumeikan University, Kyoto, Japan

**Keywords:** adaptation/constancy, color, lightness/brightness, natural image statistics

## Abstract

Kitaoka’s Tomato is a color illusion in which a semitransparent blue-green field is placed on top of a red object (a tomato). The tomato appears red even though the pixels would appear green if viewed in isolation. We show that this phenomenon can be explained by a high-pass filter and by histogram equalization. The results suggest that this illusion does not require complex inferences about color constancy; rather, the tomato’s *red* is available in the physical stimulus at the appropriate spatial scale and dynamic range.

## Introduction

**Figure 1.**
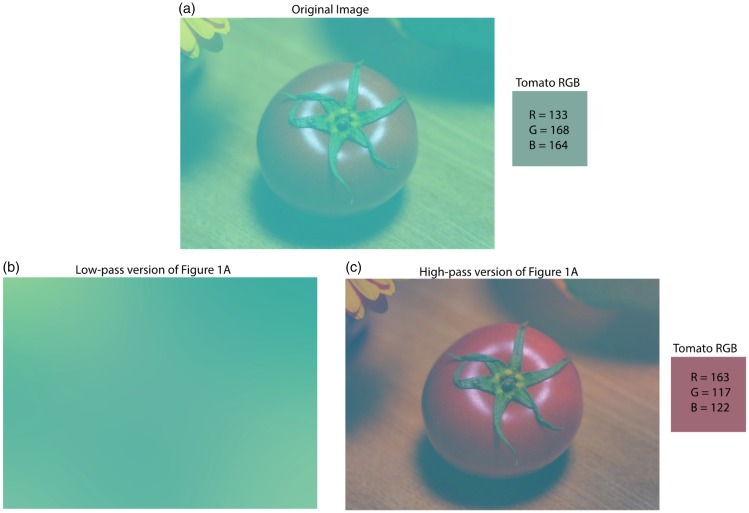
The original Kitaoka Tomato Illusion. (a) The image of the tomato is composed of green pixels. The image is decomposed into low-pass (b) and high-pass (c) images. The low-pass image contains the information of the overlay/illuminant and the high-pass image contains information from the object. The values of the pixels are shown in squares and were taken using the 1 × 1 pixel grabber in Adobe Photoshop at the same location in both images.

A color illusion created by Akiyoshi Kitaoka recently went viral on the Internet (see Figure 1). The image, reminiscent of demonstrations by Land (1959), consists of an object (a strawberry or a tomato) behind a veiling transparent layer. The image is considered an “illusion” because the tomato appears red, but the pixels that make up the tomato have greater values for B and G than for R. So, if a small patch of the tomato is viewed in isolation, the patch will appear blue-green.

The standard account for recent color phenomena such as the color-changing dress is based on color constancy (Gegenfurtner, Bloj, & Toscani, 2015; Hesslinger & Carbon, 2016; Toscani, Gegenfurtner, & Doerschner, 2017; Wallisch, 2017; Witzel, Racey, & O'Regan, 2017) and has also been applied to Kitaoka’s Tomato. Color constancy refers to the observation that objects maintain a relatively stable color appearance across a wide range of illuminants. For example, a red tomato viewed under a greenish light and then a yellowish light appears to be red under both illuminants, but the light reflected from the tomato under the greenish illuminant to the eye is wildly different from the light reflected from the tomato under the yellowish illuminant to the eye. According to the standard account of color constancy, the change in illuminant tends not to affect our perception of the object because our perceptual system forms a representation of the tomato’s material (i.e., the distal object), not a representation of the light reaching the eye (i.e., the proximal stimulus).

The problem is that the visual system does not have direct access to the material and therefore must construct a representation from responses to the light reaching the eye. Most current color constancy theories propose that the visual system forms a representation of the material’s surface based on cues in the image about the reflectance of the material and the nature of the illuminant (Foster, 2011; Lee & Smithson, 2016; Radonjic, Cottaris, & Brainard, 2015; Witzel, van Alphen, Godau, & O’Regan, 2016; Xiao, 2016). The visual system assigns these cues probabilistic weights based on prior experiences with the object, the illumination, and other information. So, in Figure 1(a), the standard color constancy account suggests that the tomato appears red because the observer has previous experience with tomatoes and with greenish illumination; the visual system automatically discounts the veiling illumination so that the observer can infer the likely color of the material—hence, the tomato appears the color of the surface (i.e., red) and not the color of the light reaching the eye (i.e., blue-green).

While inference-based approaches have their appeal, there is a long history of other approaches to such phenomena that are based on early-stage filters and on visual adaptation (Blakeslee, Cope, & McCourt, 2016; Blakeslee & McCourt, 2011; Ekroll & Faul, 2009; Hering, 1905/1964; Kingdom, McCourt, & Blakeslee, 1997; Land, 1986; McCann, Parraman, & Rizzi, 2014; Ratliff, Knight, & Graham, 1969). Indeed, many well-known illusions, like Adelson’s Checker-Shadow and Lotto and Purves’ Rubik’s cube, can easily be explained by simple computations on the image (Dixon & Shapiro, 2017; A. Shapiro & Lu, 2011) but are still given as examples of processes based on inferences, experience, and cognitive strategies.

Here, we illustrate how two low-level approaches can account for Kitaoka’s Tomato and can possibly give insight into early visual processes. The results suggest that many aspects of color and brightness illusions arise because of information physically available in the image, and that this information could potentially be extracted by processes in the early visual system.

## Demonstrations

We will demonstrate two image-processing algorithms on Kitaoka’s Tomato: One procedure is based on separating the image into components with different spatial responses (Dixon & Shapiro, 2017; A. Shapiro & Lu, 2011) and the other is based on optimal tuning with histogram equalization (see Barlow & Foldiak, 1989; A. Shapiro & Lu, 2011; A. G. Shapiro, Beere, & Zaidi, 2003; von der Twer & MacLeod, 2001; Webster, 2015; Zaidi & Shapiro, 1993). We present the demonstrations using commercial filters available in Adobe Photoshop to illustrate the simplicity of the approach and so that other investigators can test the techniques with minimal effort.

### Demonstration 1: High Spatial Frequency and Low Spatial Frequency Color Vision

Demonstration 1 is based on that idea that visual images carry information at a variety of spatial scales (Graham, 1989). Following Dixon and Shapiro (2017), we divide Kitaoka’s Tomato into low and high spatial frequency component images. Figure 1(a) shows a reproduction of the original Kitaoka Tomato created by placing an image of a tomato on Layer 1 and a semitransparent blue-green on the layer above (see Dixon & Shapiro, 2017). When these layers are combined, a target pixel on the tomato has an R, G, and B value of 133, 168, and 164, respectively (the values of B and G are higher than R); these values can be seen in the colored square next to Figure 1(a).

The low-pass and high-pass versions of Figure 1(a) are shown in Figure 1(b) and (c), respectively. To create Figure 1(b), the image in Figure 1(a) is blurred (low-pass filter) with a radius equal to 200 pixels. To create Figure 1(c), Figure 1(a) is filtered with Adobe Photoshop’s high-pass filter with a cutoff of 200.

The low-pass component (Figure 1(b)) shows a solid field with a chromaticity that approximately equals the chromaticity of the overlay (an R, G, and B value of 101, 178, and 168, respectively). The tomato is not discernable in the image; a visual system that has only a low spatial frequency response would encode global changes but would be blind to visual objects in the scene. A high-pass component (Figure 1(c)) shows the tomato as if the veiling blue-green layer has been removed. The tomato in Figure 1(c) has a stronger R value than B and G (the test pixel has an R, G, and B value of 163, 117, and 122, respectively)—corresponding more directly to an observer’s reports. A high-pass filter, therefore, is equivalent to subtracting the blurred image from the original and adding a constant. In effect, Figure 1(c) “discounts” the information contained in the blurred image (Figure 1(b)) from the original image (Figure 1(a)).

### Demonstration 2: Histogram Equalization

The visual system continually adapts to chromatic and luminance information in the environment. Historically, and in many current Perception textbooks, adaptation is discussed in terms of “fatigue”: a process in which a cell lowers its response rate to steady stimulation. However, as a general rule, visual adaptation can be considered a process for maximizing the response range available to the visual system (see Barlow & Foldiak, 1989; Craik, 1938; Webster, 2015). For example, when looking at a field of green grass, the visual system should adjust its response so that it can discriminate the maximum number of shades of green, at the expense of discrimination of shades of red. So, if an image has a statistical distribution along a particular dimension, the visual system should adjust its response so that it can maximize the number of levels that can be discriminated along that dimension. One way of encapsulating this principle is with histogram equalization, a standard image-processing technique.

**Figure 2.**
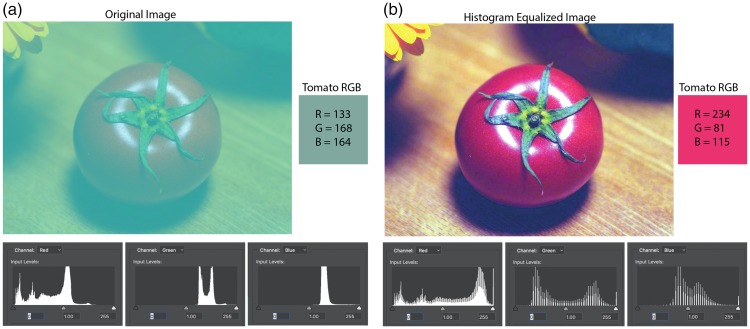
The original Kitaoka Tomato Illusion showing (a) the distribution of the R, G, and B pixels in the image and (b) the image adjusted to equalize the distribution of each of the pixels.

Here, we will apply histogram equalization to Kitaoka’s Tomato. Figure 2(a) shows the original image along with the histogram of the R, G, and B values. Figure 2(b) shows the image after a rough equalization correction. The histogram equalization was performed manually using a level operator and adjusting the maximum and minimum of each channel independently. A simple histogram equalization technique eliminates the effect of the overlay and returns an image close to the original image. For Figure 2(b), the readjusted range for the R value is a maximum of 158 and minimum 4; G, 205 and 150; B, 240 and 32. The R, G, and B values of the test patch are now 234, 81, and 115, respectively.

The result shows that at the appropriate dynamic range, the tomato is red—a result that should be expected given the results from Figure 1(b), since the histogram equalization procedure recenters the average value of the image, thereby eliminating the blue-green offset. In effect, the procedure recenters each channel, a principle otherwise referred to as von Kries’ adaptation (1905/1970).

## Conclusion

Kitaoka’s Tomato is a strong color illusion, and variants of the illusion have become popular on the Internet. We have shown two methods for eliminating the illuminant based purely on the stimulus and without any consideration of the material properties of the object. Numerous filter-based models would handily account for Kitaoka’s Tomato. For instance, the Milano Retinex Family (Rizzi & Bonanomi, 2017) shows variations of Land’s (1983, 1986) Retinex algorithms. Almost all of these algorithms could account for the illusions, as could filter-based models such as Blakeslee and McCourt (1999, 2001, 2004), Robinson, Hammon, and de Sa (2007), Dakin and Bex (2003), Jobson, Rahman, and Woodell (1997), Zeman, Brooks, and Ghebreab (2015), and Buchsbaum (1980). Indeed, even von Kries’ adaptation (1905/1970) would likely remove the average value of the background.

The methods presented here differ from other filtering or adaptation models only in simplicity. Following A. Shapiro and Lu (2011), we are suggesting that the filters “work” in part because the information for color constancy (and for estimating the illuminant) exists in the stimulus at the appropriate spatial/intensity scale. That is, the overlay changes the chromaticity of the tomato at the level of the pixel, but not for high spatial frequency content. Similarly, as demonstrated by Dixon and Shapiro (2017), global changes in illumination are primarily carried in the low spatial frequency content. Most filtering or adaptation models therefore will be successful or differ from each other in how they adjust to extract that information from the image and how they equalize the dynamic range of the responding color channels.

Demonstrations of the simplicity and efficacy of simple filter approaches are necessary because recent publications, expert discussion on the internet and news, and discussions on the CVNet mailing list seem to ignore the power of these basic approaches in favor of models based on Bayesian priors and sophisticated estimates concerning our knowledge about the material properties and illumination. In the standard color constancy model, the light reaching the eye is almost incidental to our perception since our perception is assumed to care primarily about understanding the properties of a distal object. It therefore needs to be reemphasized that very simple computational operations can give a first approximation to these distal properties under many circumstances.

Low-level processes for color constancy are often dismissed because they seem to lack a functional purpose, because they cannot account for *all* brightness/lightness phenomena (such as assimilation), and because they are thought to produce “scalloped” artifacts that are not typically perceived. We are not suggesting that the simple operations capture exactly what the visual system does, nor do we think that this is a complete model of the visual system. Furthermore, the operations in Demonstrations 1 and 2 are done to the whole image, and such operations (if they exist) are almost certainly accomplished by local processes. Also, as we have noted elsewhere, our one parameter filter models have difficulty with Cornsweet edges (A. Shapiro & Lu, 2011) and some gradients (Dixon & Shapiro, 2017, Figure 9) and many versions of White’s effect and assimilation.

However, it would be surprising if the principles underlying these filters did not exist in some analogous physiological form. An easy way to implement a tunable spatial filter (as in Figure 1(c)) would be with an array of Difference of Gaussian filters, where the radii of the center and surround Gaussians can adapt independently of each other. The size of the inhibitory surround controls the amount of low spatial frequency content passed by the filter, and the size of an excitatory center affects the amount of high spatial frequency content passed by the filter. Indeed, the simplicity of such a tunable system suggests a purpose for why center–surround receptive fields are found in retinal cells and are ubiquitous in nearly all sensory systems.

The filter, however, does not have to occur at an early retinal stage since, as was emphasized by A. Shapiro and Lu (2011), a cortical representation of an object is itself a form of high-pass filter. Presumably, the early visual system samples the retinal image at a range of spatial scales; the later visual system builds representations of objects by selectively pooling from these filtered responses. The pooling processes would give a higher weight to filters that respond maximally to regions that are about the same size of the object and would give a lower weight to filters that respond maximally to areas larger than the object. This process diminishes the importance of the low spatial content and therefore acts something like the processes that create Figures 1(c) and 2(b). More than that, since global illumination is primarily contained in the low spatial frequency content, any representation of an object will not encode information about the illuminant and will intrinsically behave with some level of color constancy.
